# New Strategy for Improving the Accuracy of Aircraft Positioning Based on GPS SPP Solution

**DOI:** 10.3390/s20174921

**Published:** 2020-08-31

**Authors:** Kamil Krasuski, Adam Ciećko, Mieczysław Bakuła, Damian Wierzbicki

**Affiliations:** 1 Institute of Navigation, Military University of Aviation, 08-521 Dęblin, Poland; mieczyslaw.bakula@uwm.edu.pl; 2 Faculty of Geoengineering, University of Warmia and Mazury in Olsztyn, 10-720 Olsztyn, Poland; a.ciecko@uwm.edu.pl; 3 Institute of Geospatial Engineering and Geodesy, Faculty of Civil Engineering and Geodesy, Military University of Technology, 00-908 Warszawa, Poland; damian.wierzbicki@wat.edu.pl

**Keywords:** GPS, SPP method, weighted mean method, accuracy, RMS error

## Abstract

The paper describes and presents a new calculation strategy for the determination of the aircraft’s resultant position using the GPS (Global Positioning System) SPP (Single Point Positioning) code method. The paper developed a concept of using the weighted average model with the use of measuring weights to improve the quality of determination of the coordinates and accuracy of GPS SPP positioning. In this research, measurement weights were used as a function of the number of GPS satellites being tracked, and geometric PDOP (Position Dilution of Precision) coefficient. The calculations were made using navigation data recorded by two independent GPS receivers: Thales Mobile Mapper and Topcon HiPerPro. On the basis of the obtained results, it was found that the RMS (Root Mean Square) accuracy of positioning for XYZ geocentric coordinates was better than 1.2% to 33.7% for the weighted average method compared to a single GPS SPP solution. The proposed approach is therefore of practical application in air navigation to improve the quality of aircraft positioning.

## 1. Introduction

In the 21st century, the GNSS (Global Navigation Satellite System) satellite technology has become a common method of aircraft positioning in the field of air navigation and air transport [1,2,3,4]. The framework of the application, operation, and implementation of GNSS satellite technology in aviation has been clearly defined by the International Civil Aviation Organization (ICAO) [5]. Annex 10 to the Chicago Convention currently allows the use of the GNSS satellite technique in aviation for the purpose of air operations within the systems:

GPS and GLONASS (Globalnaya Navigacionaya Sputnikovaya Sistema) as GNSS autonomous navigation systems, certified by the ICAO.

ABAS (aircraft based augmentation system), SBAS (satellite based augmentation system), GBAS (ground based augmentation system) as augmentation systems, certified by the ICAO [6].

In the case of GNSS systems, only GPS and GLONASS are certified for general use in civil aviation. It should be emphasized that the GPS and GLONASS systems are fully operational and provide continuous satellite positioning for their users around the globe, 24 h a day. The certification of the GPS and GLONASS navigation systems in civil aviation includes parameters such as accuracy, availability, integrity, and continuity. In accordance with the ICAO recommendation, the accuracy of determining the position of an aircraft with the use of a GPS navigation system must not exceed 17 m for navigation in the horizontal plane and 37 m for the vertical component, respectively, for all air operations across the globe [7]. The accuracy of aircraft position is estimated as the difference between the current coordinates and reference position of aircraft. In addition, the ICAO recommendation on accuracy refers to the SPP (single point positioning) method in the GPS system.

The SPP method used in the air navigation system utilizes absolute positioning using code observations. The SPP code method has been repeatedly used in flight tests in order to determine the accuracy of aircraft positioning by means of the GPS satellite system [8]. In air navigation, the positioning accuracy of the SPP method is designated in relation to the reference solution based on the differential DGPS (differential GPS) technique, the differential DD (double difference) technique or the RTK-OTF (real time kinematic-on the fly) differential technique. Within the DGPS differential technique, the aircraft position is determined using code differential corrections, transmitted from the GNSS reference station to a mobile receiver, mounted on board an aircraft. In the differential DD solution, the reference position of the aircraft is determined by means of phase observations in the post-processing mode. On the other hand, in the differential RTK-OTF solution, the reference position of the aircraft is determined using phase observations in real time. 

Studies on aircraft positioning accuracy using the SPP code method have been conducted worldwide in aeronautical experiments and described in scientific papers. The articles [9,10] describe the results of the aerial test performed in Florida, USA, in 1994. The position of the aircraft was determined using GPS observations in the SPP code method. The accuracy of aircraft positioning using the SPP code method was referred to the DGPS solution. The consistency between the SPP and DGPS mode was about 1 m (RMS) in latitude and longitude, and 2 m (RMS) in height, with instantaneous errors of up to a few meters. IGS (International GNSS Service) products, mainly precision orbits and GPS satellite clocks, were used in the calculations to improve aircraft positioning accuracy. In turn, the authors in [11] presented the results of research carried out in Greenland in 2000. The position of the aircraft was determined using the GPS SPP code method. The result of aircraft positioning from the SPP code method was referred to the DD solution. The accuracy in the form of RMS error was up to ±1.5 m in the horizontal plane and up to ±1 m for the vertical component. IGS products such as precision orbits and clocks of GPS satellites, were used in the calculations. Another article [12] contained the results of the research that took place in San Jose, CA, USA. In this case, the position of the plane was determined by the SPP code method using GPS/GLONASS observations. The result of the plane’s positioning using the SPP method was referred to the DD solution using phase GPS/GLONASS observations. The accuracy was in the range of ±1 to ±5 m in the horizontal plane and up to ±10 m for the vertical component. Other papers [13,14] contained a description and the results of the aerial test performed in Halifax, Nova Scotia, Canada, in 2004. In this test, the position of aircraft was determined by the SPP code method using GPS only observations and compared to the DD solution. The accuracy was up to ±1.5 m in the horizontal plane and up to ±3 m for height. In this research, the IGS products, mainly precision orbits and clocks of GPS satellites and the ionosphere model in IONEX format, were used in the calculations in order to increase the positioning accuracy of the aircraft. The aerial experiments were also carried out in Europe. The research from the Netherlands performed in 2005 is presented in [15]. In this example, the position of the plane was determined using GPS observations and the SPP code method. The accuracy of aircraft positioning from the SPP code method was referred to the DD solution. The accuracy achieved was very good, reaching about ±0.5 m in the horizontal plane and about ±1.5 m for height. Such a high accuracy was possible thanks to the use of IGS products (i.e., precision GPS orbits and clocks, ANTEX format for precision antenna parameters as well as estimation of differential code biases (DCB) and instrumental errors), which were used in the calculations to improve aircraft positioning accuracy.

In Poland, most of the aeronautical experiments using GNSS satellite technology were carried out by the scientific team of the Department of Air Navigation (Military University of Aviation in Dęblin) as part of various research projects (e.g., “BRDA”, “ODRA”, “DUNAJ”, or “LIWIEC”). Detailed characteristics of the research conducted in Poland are presented below. The authors in [16] presented the results of research performed in Dęblin in 2000. The position of the plane was determined by the SPP method using GPS code observations. The result of aircraft positioning in the SPP code method was referred to the RTK-OTF solution. The accuracy was from ±0.5 to ±10 m in the horizontal plane and up to ±26 m for the height. Moreover, they also describe the results of research in the frame of the “DUNAJ” national aviation experiment, performed in Dęblin in 2001. The results obtained in 2001 were quite poorly accurate, ranging from ±8 to ±29 m in the horizontal plane and up to ±48 m for height. In articles [17,18,19], the results of aerial testing in Dęblin in 2003, 2007, and 2010 were presented. In all of these cases, position of the plane was determined using GPS observations within the SPP code method and referred to the RTK-OTF solution. The horizontal accuracy was up to ±3 m, ±21 m, and ±3 m for the years 2003, 2007, and 2010, respectively. The vertical accuracy was up to ±12 m, ±13.5 m, and ±8 m for years 2003, 2007, and 2010, respectively. The experiment in Dęblin also covered the examination of positioning with the use of EGNOS (European Geostationary Navigation Overlay Service) corrections in order to increase the aircraft positioning accuracy. Since the EGNOS system at that time was not fully operational and the testing area was on the edge of the coverage of Safety-of-Life (SoL) service, the results were less accurate than expected. The positioning accuracy of the EGNOS solution was up to ±19 m, ±21 m, and ±19 m in the horizontal plane and up to ±20 m ±13.5 m, and ±23 m for height for years 2003, 2007, and 2010, respectively.

The results of other Polish aerial experiments performed in Chełm in 2010 and in Mielec in 2011 were presented in [20,21]. In [20], the authors present the results of research from the aviation test carried out in 2010 in Chełm. During the tests, the position of the aircraft was determined by the GPS SPP code method. The results of the positioning of the plane with GPS SPP were referred to the RTK-OTF solution. The accuracy was up to ±4 m in the horizontal plane and up to ±5 m for height. During these tests, EGNOS corrections were also applied to investigate the accuracy of aircraft positioning using EGNOS. The positioning accuracy of the EGNOS solution was up to ±8 m in the horizontal plane and up to ±10.5 m for height. It should be noted that at that time, Chełm, located in the southeastern part of Poland, was outside the coverage of SoL service. Next, [21] describes the results of the aviation test carried out in Mielec in 2011. In these tests, the position of the aircraft was also determined using the GPS SPP code solution and its results were related to the RTK-OTF solution. In this case, the accuracy was up to ±65 m in the horizontal plane and up to ±25 m for height. The data recorded during the flight in Mielec in 2011 were also used in the study [22]. In this work, the position of the plane determined by the GPS SPP method was compared to the PPP measurement technique. The position consistency was obtained at the level of up to ±6 m in the XYZ geocentric coordinate system. Another paper [23] presented the results of GPS SPP and EGNOS positioning, which were related to RTK-OTF solution, in research from an aviation test conducted in 2013 in Dęblin. The accuracy in the horizontal plane was up to ±11 m and up to ±7 m for the SPP and EGNOS methods, respectively. Vertical accuracy was ±12 m and ±14.5 m for the SPP and EGNOS methods, respectively. The results of aeronautical tests performed in Dęblin in 2016 are presented in [24]. In this case, the position of the plane was determined by the GPS SPP code method and the consistency of results to the DGPS solution was up to ±11 m in the horizontal plane and up to ±26 m for the vertical component.

On the basis of the literature on the subject of the research, it can be concluded that the problem of optimal strategy for solving the position of an aircraft using the GPS SPP code method is still relevant. Moreover, the aspect of improving positioning accuracy is apparent. The authors of the article decided to carry out scientific research and necessary analyses concerning the subject of improving the quality of GPS SPP positioning in air navigation, thus increasing the accuracy of the coordinates of the aircraft. The aim of the paper was to present a new strategy for determining the position of an aircraft using the SPP code method leading to the improvement of GPS SPP positioning accuracy in air navigation. The paper proposes the use of the weighted average method to determine the resultant coordinates of the aircraft based on navigation data from two independent GPS satellite receivers. In this work, the measurement weights as a function of the number of GPS satellites being tracked and the geometric PDOP coefficient were used. The finally determined coordinates of the aircraft were compared with the reference trajectory of the flight calculated by the differential RTK-OTF technique. The applied weighted average method significantly improves the accuracy of aircraft positioning in relation to a classical GPS SPP solution.

The rest of the paper is structured as follows. Section 2 presents the test data and methodology, Section 3 describes the experiments performed, Section 4 discusses the results in detail, and finally, Section 5 provides a brief summary and the conclusions of this work.

## 2. Materials and Methods

The GNSS module in the aircraft avionics shall be equipped with at least two GPS satellite receivers for determining the navigation parameters of the aircraft during flight. In order to cross-check the obtained coordinates of the aircraft position, a minimum number of two receivers is needed to ensure at least one degree of freedom (n−1, n−number of GPS receivers). In the case under consideration, the number of degrees of freedom was 1, so the measurement was redundant. The resultant position of the aircraft will not be affected by systematic and accidental errors from a single receiver only. Therefore, determination of the resultant position of the aircraft will reduce measurement errors in the analyzed test. Using two GPS receivers is a minimum but sufficient number of on-board sensors in aeronautical tests in order to determine the aircraft’s resultant position. 

Each GPS receiver shall determine the position of the aircraft using the SPP code method algorithm. This configuration makes it possible to use a GPS positioning scheme based on a weighted average model for navigation data. In this case, the navigation data are mainly related to the components of the plane’s position in the three-dimensional XYZ ortho-Cartesian global coordinate system. Then, the mathematical model of determining the resultant position of an aircraft based on the SPP code method for the weighted average algorithm can be written as follows [25]:

(1) $$\{\begin{array}{c} X_{GPS}^{m}=\frac{\sum X_{GPS}^{i}\cdot w_{i}}{\sum w_{i}}\\ Y_{GPS}^{m}=\frac{\sum Y_{GPS}^{i}\cdot w_{i}}{\sum w_{i}}\\ Z_{GPS}^{m}=\frac{\sum Z_{GPS}^{i}\cdot w_{i}}{\sum w_{i}} \end{array}$$

where$\;\left(X_{GPS}^{m},Y_{GPS}^{m},Z_{GPS}^{m}\right)$ is the resultant position of the aircraft, overall weighted average based on the GPS SPP solution;$\;w_{i}$ is the measurement weight;$\;\left(X_{GPS}^{i},Y_{GPS}^{i},Z_{GPS}^{i}\right)$ is the position of the aircraft determined from a single GPS SPP solution; and i is the number of a single GPS SPP solution for each receiver, i=1,2.

By taking into account the receiver indexes (i=1,2), Equation (1) can be converted to the form:

(2) $$\{\begin{array}{c} X_{GPS}^{m}=\frac{X_{GPS}^{1}\cdot w_{1}+X_{GPS}^{2}\cdot w_{2}}{w_{1}+w_{2}}\\ Y_{GPS}^{m}=\frac{Y_{GPS}^{1}\cdot w_{1}+Y_{GPS}^{2}\cdot w_{2}}{w_{1}+w_{2}}\\ Z_{GPS}^{m}=\frac{Z_{GPS}^{1}\cdot w_{1}+Z_{GPS}^{2}\cdot w_{2}}{w_{1}+w_{2}} \end{array}$$

Whereas the measurement weight parameter $w_{i}$ can be expressed with the following mathematical formulas:

(3) $$w_{i}=\frac{1}{ns_{i}}\;\;\;\;\;\;\;\;or\;\;\;\;\;\;w_{i}=\frac{1}{PDOP_{i}}$$

where$\;ns_{i}$ is the number of GPS satellites being tracked by each receiver; and$\;PDOP_{i}$ is the value of the 3D position PDOP (position dilution of precision) for each GPS receiver.

By substituting the weighting parameters from Equation (3) to Equation (2), we finally obtain:

Case I:

(4) $$\{\begin{array}{c} X_{GPS}^{m}=\frac{X_{GPS}^{1}\cdot \frac{1}{ns_{1}}+X_{GPS}^{2}\cdot \frac{1}{ns_{2}}}{\frac{1}{ns_{1}}+\frac{1}{ns_{2}}}\\ Y_{GPS}^{m}=\frac{Y_{GPS}^{1}\cdot \frac{1}{ns_{1}}+Y_{GPS}^{2}\cdot \frac{1}{ns_{2}}}{\frac{1}{ns_{1}}+\frac{1}{ns_{2}}}\\ Z_{GPS}^{m}=\frac{Z_{GPS}^{1}\cdot \frac{1}{ns_{1}}+Z_{GPS}^{2}\cdot \frac{1}{ns_{2}}}{\frac{1}{ns_{1}}+\frac{1}{ns_{2}}} \end{array}$$

Case II:

(5) $$\{\begin{array}{c} X_{GPS}^{m}=\frac{X_{GPS}^{1}\cdot \frac{1}{PDOP_{1}}+X_{GPS}^{2}\cdot \frac{1}{PDOP_{2}}}{\frac{1}{PDOP_{1}}+\frac{1}{PDOP_{2}}}\\ Y_{GPS}^{m}=\frac{Y_{GPS}^{1}\cdot \frac{1}{PDOP_{1}}+Y_{GPS}^{2}\cdot \frac{1}{PDOP_{2}}}{\frac{1}{PDOP_{1}}+\frac{1}{PDOP_{2}}}\\ Z_{GPS}^{m}=\frac{Z_{GPS}^{1}\cdot \frac{1}{PDOP_{1}}+Z_{GPS}^{2}\cdot \frac{1}{PDOP_{2}}}{\frac{1}{PDOP_{1}}+\frac{1}{PDOP_{2}}} \end{array}$$

In the general matrix notation, the proposed methodology can be written as follows:

(6) $$Q=\frac{A}{B}=\frac{\sum R_{GPS}^{i}\cdot w_{i}}{\sum w_{i}}$$

where Q is the resulting position of the aircraft, $Q=\left[\begin{array}{c} X_{GPS}^{m}\\ Y_{GPS}^{m}\\ Z_{GPS}^{m} \end{array}\right]$; A is the numerator of the mathematical expression in Equation (6), $A=\sum R_{GPS}^{i}\cdot w_{i}$;$\;R_{GPS}^{i}$ is the coordinates of the aircraft position for each GPS receiver separately, $R_{GPS}^{i}=\left[\begin{array}{c} X_{GPS}^{i}\\ Y_{GPS}^{i}\\ Z_{GPS}^{i} \end{array}\right]$; and B is the denominator of the mathematical expression in Equation (6), $B=\sum w_{i}$.

The resultant coordinates of the aircraft’s position are determined according to Equations (4)–(6) using different weight models. The calculation strategy presented in the research methodology will determine which weight model is the best for the weighted average method. It seems even more important for the presented calculation strategy to determine the positioning accuracy for the weighted average method. The best match of the obtained resultant coordinates with respect to the flight reference trajectory will show which weight model is optimal for solving the position from the GPS SPP code method. As part of the accuracy analysis, position errors and root mean square (RMS) errors were determined. Position errors represent matching of resultant coordinates with respect to the flight reference trajectory and are calculated according to the relationship [22,26]:

(7) $$\{\begin{array}{c} DX=X_{GPS}^{m}-X_{ref}\\ DY=Y_{GPS}^{m}-Y_{ref}\\ DZ=Z_{GPS}^{m}-Z_{ref} \end{array}$$

where (DX,DY,DZ) is the positioning errors; and $\left(X_{ref},Y_{ref},Z_{ref}\right)$ is the reference position of the aircraft determined by the differential RTK-OTF technique.

In the next step, the RMS error is calculated according to [27]:

(8) $$\{\begin{array}{c} RMSdX=\sqrt{\frac{\left[DX^{2}\right]}{N}}\\ RMSdY=\sqrt{\frac{\left[DY^{2}\right]}{N}}\\ RMSdZ=\sqrt{\frac{\left[DZ^{2}\right]}{N}} \end{array}$$

where (RMSdX,RMSdY,RMSdZ) is the RMS error along the XYZ axis; and N is the number of position records, number of measurement epochs.

## 3. Research Test

A number of tests and calculations were performed to determine the accuracy of aircraft positioning and to assess the usefulness of the proposed computing strategy. The calculations used both real-time and post-processing aircraft position navigation data. All research materials were taken from the flight test performed by the Cessna 172 aircraft, in the vicinity of the military aerodrome EPDE (European Poland Deblin) in Dęblin [26].

**Figure 1:**
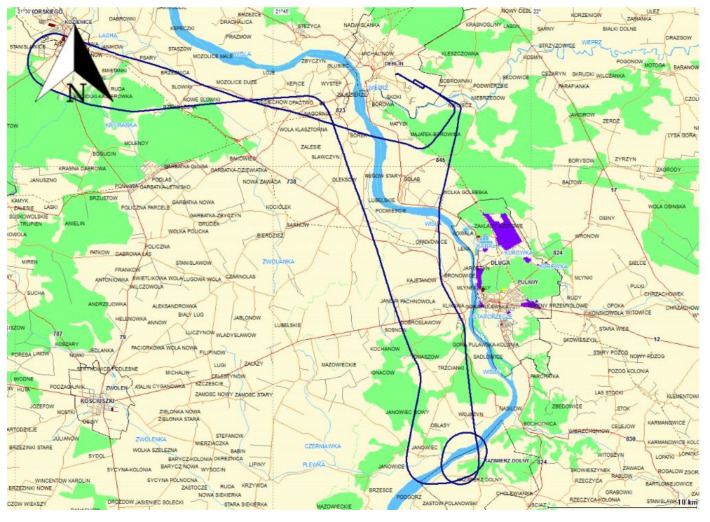
The trajectory of aircraft in horizontal plane.

The flight route of the Cessna 172 passed over the following locations: Dęblin–Kozienice–Kazimierz Dolny–Puławy–Dęblin. The flight test lasted from 09:39:03 to 10:35:03 according to GPST (GPS time). Figure 1 shows the trajectory of the Cessna 172 aircraft in the horizontal plane. It should be noted that for the results presented in the paper, the weighted average model was used for the speed range from 0 to 80 m/s [28].

To determine the precise state of the troposphere during experimental flight, meteorological data from a short TAF (terminal aerodrome forecast) report were used. For military airports, a special format of meteorological data called TAF has been developed, which enables weather forecasting for a given airport. The TAF report was generated and downloaded from the OGIMET service website [29]. It should be mentioned that the short TAF report was updated for EPDE (European Poland Deblin) airport at 05:00, 08:00, and 11:00 o’clock UTC. Detailed attention was given to a fragment of the TAF report specifying the meteorological conditions from 08:00 o’clock UTC. At this time, the following information was given: the forecast average wind direction was 330° with an average speed of 10KT; the forecast visibility was about 8000 m, limited by weak rainfall; the lowest cloud layer with a cloud cover of 3–4/8th degree with a cloud base of approximately 800 ft (250 m), another cloud layer with a cloud cover of 5–7/8th degree with a cloud base of 1700 ft (520 m), and the highest cloud layer with a cloud cover of 8/8th degree with a cloud base of approximately 7000 ft (2100 m). Furthermore, between 09:00 and 15:00 UTC time, the possibility of periodical changes of visibility and cloud cover up to 4000 m as well as passing rainfall was predicted with 30% probability.

For the needs of precise positioning of the Cessna 172 aircraft, two satellite receivers were mounted on board: the Thales Mobile Mapper and Topcon HiPerPro [28]. The Thales Mobile Mapper recorded the position of the Cessna 172 in real time based on the SPP solution using a GPS system. In turn, the Topcon HiPerPro receiver collected raw GPS observations for post-processing calculations. Both GNSS satellite receivers were mounted in the cockpit of the Cessna 172, behind the Plexiglass. In addition, the GPS code and phase measurements recorded by the Topcon HiPerPro receiver were used for post-processing calculations in the RTKLIB v.2.4.3 software, available at http://rtklib.com [30]. The RTKLIB program also reproduced the position of the Cessna 172 airplane according to the SPP code method algorithm.

During the test on the Cessna 172, the Thales Mobile Mapper receiver was configured as follows [26]:

- the receiver’s internal software: Mobile Mapper and Mobile Mapper Office;
- data export format: SHP (shapefile), MIF (MapInfo Interchange Format), and DXF (data exchange format file);
- coordinate system: global, WGS-84 as a standard;
- calculation mode: GPS L1;
- final coordinates format: ellipsoidal coordinates and geocentric coordinates;
- maximum number of GPS satellites tracked: 12 GPS satellites;
- GPS satellite tracking: sequential;
- interval of calculations and recording: 1 s;
- positioning accuracy: up to 3 m with a confidence level of 0.95;
- mathematical positioning model: based on SPP code method;
- positioning mode: real time;
- GPS ephemeris data: on-board ephemeris, broadcast in real-time;
- sources of GPS code measurement errors: based on data from on-board navigational message (Kepler orbit model, satellite clock error, Klobuchar ionosphere model, relativistic correction, timing group delay (TGD));
- weighting of the measurement results: applied;
- satellites elevation mask: 5 degrees; and
- reference time: GPST.

On the other hand, during the numerical calculations in post-processing mode based on the data from the Topcon HiPerPro receiver, the configuration of the RTKLIB software was as follows [26]:

- source of GPS observation data: RINEX 2.11 file;
- coordinate system: WGS-84;
- calculation mode: GPS L1/L2;
- final coordinates format: ellipsoidal coordinates and geocentric coordinates;
- interval of calculations: 1 s;
- positioning mode: post-processing,
- source of GPS satellites ephemeris: GPS navigation data message, broadcast in real-time;
- sources of GPS code measurement errors: based on data from on-board navigational message (Kepler orbit model, satellite clock error, Klobuchar ionosphere model, relativistic correction, TGD, Saastamoinen troposphere model);
- weighting of the measurement results: applied,
- satellites elevation mask: 5 degrees;
- reference time: GPST.

Eventually, the XYZ coordinates obtained from the two independent GNSS receivers were used in the mathematical scheme presented in (1−7) to determine the resulting position of the aircraft. In this step, the calculations were performed in Scilab v.6.0.0 software [31]. The results of the numerical analysis from Scilab are presented in Section 4 of this work.

## 4. Results

**Figure 2:**
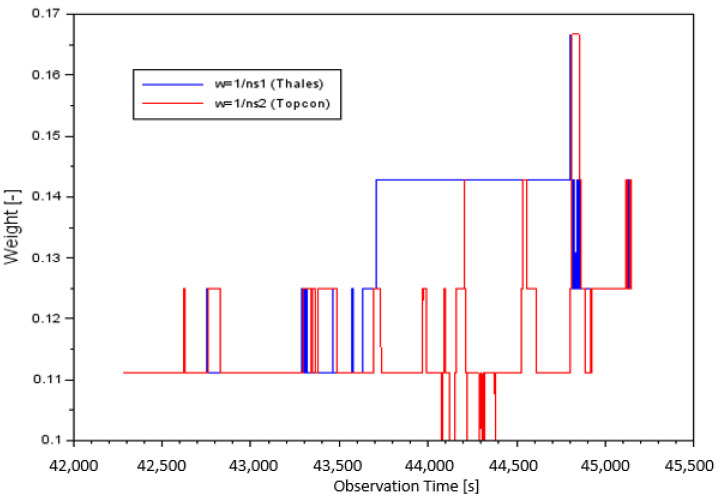
The weight value for Case I: Thales (blue) and Topcon (red).

In the first stage of the analysis of the test results, the values of the obtained measurement weights for Case I and Case II are presented according to Equations (4) and (5). Figure 2 shows the measurement weights as a function of the number of GPS satellites tracked by Thales Mobile Mapper in blue and Topcon HiPerPro in red. 

The values of the measurement weights for Case I, depending on number of satellites tracked, for Equation (4), for parameter $w=\frac{1}{ns_{1}}$ (based on Thales receiver) was from 0.111 to 0.166. In addition, the mean value of the measurement weight $w=\frac{1}{ns_{1}}$ equaled 0.126 and the median was 0.125. On the other hand, the values of the measurement weights for parameter $w=\frac{1}{ns_{2}}$ (based on the Topcon receiver) were from 0.100 to 0.166. In this case, the mean value of the measurement weight $w=\frac{1}{ns_{2}}\;$ was 0.116 and the median was 0.111.

**Figure 3:**
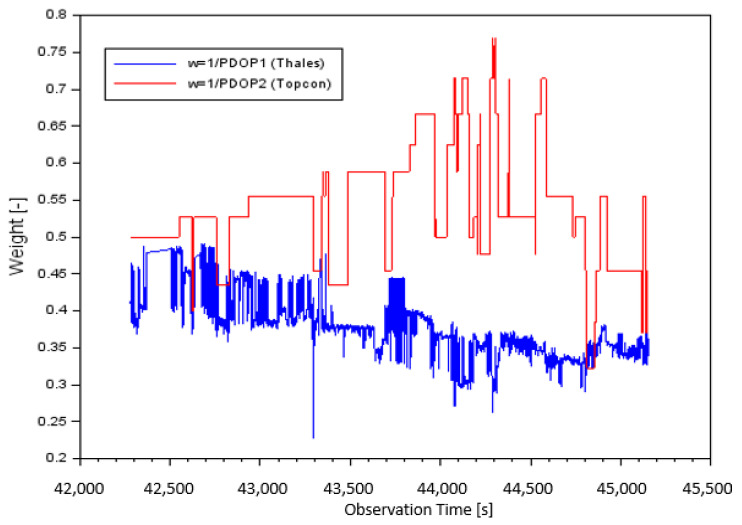
The weight value for Case II: Thales (blue) and Topcon (red).

Figure 3 presents the measurement weights as a function of PDOP value separately for the Thales Mobile Mapper (blue) and Topcon HiPerPro (red) receivers. The values of the measurement weights for Case II, depending on PDOP value, for Equation (5), for parameter $w=\frac{1}{PDOP_{1}}$ (based on Thales receiver) was from 0.228 to 0.490. Moreover, the mean value of measurement weight $w=\frac{1}{PDOP_{1}}$ was equal to 0.375 and the median was 0.372. The values of the measurement weights for parameter $w=\frac{1}{PDOP_{2}}$ (based on Topcon receiver) were from 0.323 to 0.769. The mean value of the measurement weight $w=\frac{1}{PDOP_{2}}$ was equal to 0.538 and the median was 0.526.

When selecting the weighting factors $w_{i}$, the authors wanted to show their influence as a function of observation time. Therefore, the statistical analysis of the weighting factors as well as the determination of the dispersion of results, the arithmetic mean and the median was very important. In the research, all the results of the individual weighting factors to determine the correct accuracy values were used. Of course, to determine the average results of the parameters (DX,DY,DZ), it would be efficient to use the arithmetic mean alone out of the individual weighting factors. However, for the evaluation of the overall test, it is believed that the use of all the results of the weighting factors was necessary. 

**Figure 4:**
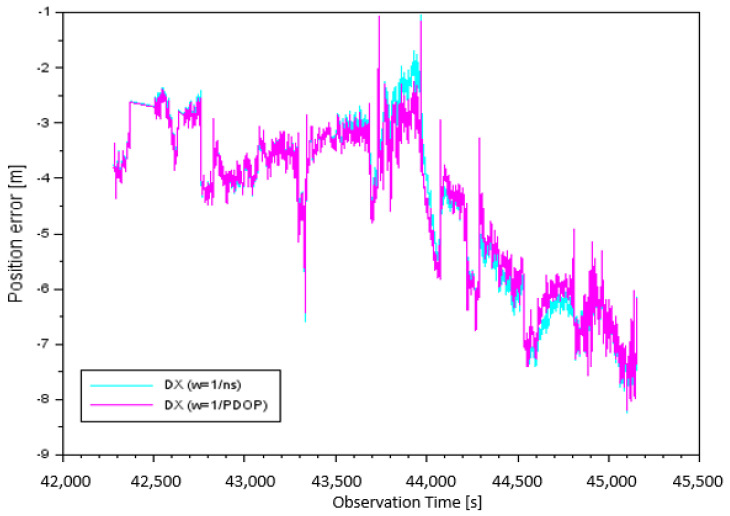
Position error along the X-axis.

In the next stage of the analysis of the results, position errors (DX,DY,DZ) were determined according to a mathematical expression (7). Figure 4 shows the results of the DX component taking into account the individual measurement weights. The values of DX errors for parameter $w=\frac{1}{ns}$, depending on the number of satellites tracked, were from −8.24 m to −1.03 m. It must be noted that the arithmetic mean for DX, based on the number of satellites, was equal to −4.51 m. The DX position errors for parameter $w=\frac{1}{PDOP}$, depending on PDOP, were from −8.19 m to −1.06 m. It is worth noting that in this case, the arithmetic mean of the DX component was −4.51 m.

**Figure 5:**
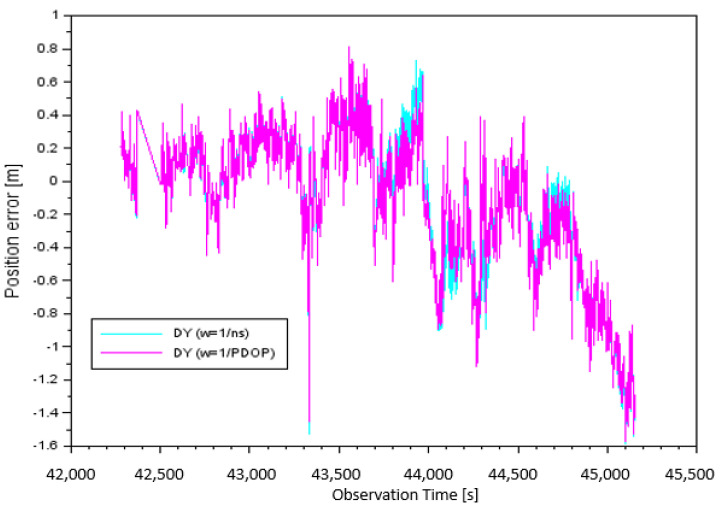
Position error along the Y-axis.

In Figure 5, the result of the DY component, taking into account the individual measurement weights, is presented. The values of the DY errors for parameter $w=\frac{1}{ns}$, depending on the number of satellites tracked, were from −1.59 m to +0.75 m. It should be noted that the arithmetic mean for the DY component, based on the number of satellites, was equal to −0.12 m. The DY position errors for the parameter $w=\frac{1}{PDOP}$, depending on PDOP, were from −1.58 m to +0.81 m. It is worth noting that in this case, the arithmetic mean of the DY component was equal to −0.14 m.

**Figure 6:**
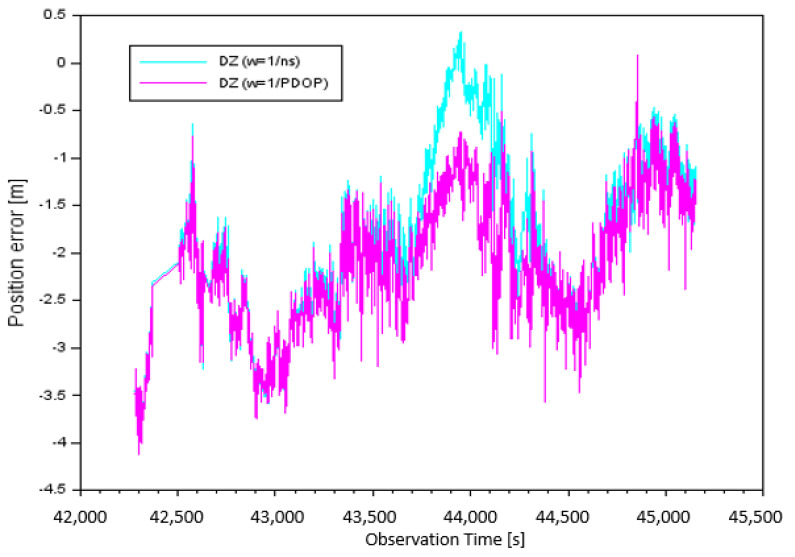
Position error along the Z-axis.

Figure 6 illustrates the results of the DZ component of position errors taking into account the individual measurement weights. The error values of DZ for parameter $w=\frac{1}{ns}$, depending on the number of satellites tracked, were from −4.06 m to +0.33 m. It should be noted that the arithmetic mean for parameter DZ was equal to −1.85 m. The error values of DZ for the parameter $w=\frac{1}{PDOP}$, depending on PDOP, were from −4.12 m to +0.09 m. It is worth mentioning that in this case, the arithmetic mean of the DZ component was equal to −2.12 m.

After determining the components of position error (DX,DY,DZ), a statistical measure of accuracy in the form of RMS value was derived using Equation (8). The accuracy values of RMS along the X-axis ranged from 4.73 m to 4.77 m, along the Y-axis from 0.44 m to 0.46 m, while the RMS along Z-axis ranged from 2.05 m to 2.23 m. It should be noted that a lower value of RMS along the X- and Y-axes was observed when the measurement weight was $w=\frac{1}{PDOP}$. The higher value of RMS along the X- and Y- axes was noticed when the measurement weight was $w=\frac{1}{ns}$. On the other hand, for the Z-axis, a lower RMS value was achieved when the measurement weight was $w=\frac{1}{ns}$, while for the measurement weight $w=\frac{1}{PDOP}$, the RMS along the Z-axis was higher. The results of the RMS values for the tested measurement weights are summarized in Table 1.

## 5. Discussion

**Figure 7:**
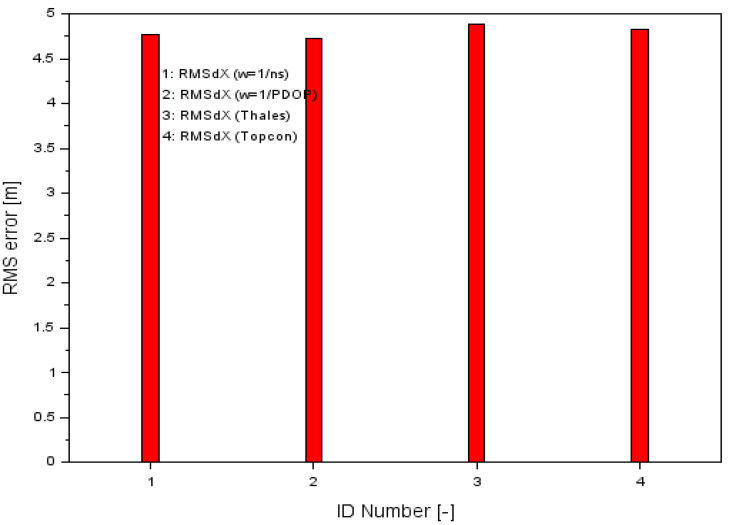
Comparison of RMS errors along the X-axis.

The presented above example proved that the proposed research method gives better results against the single GPS SPP solution in aircraft navigation. For this purpose, a comparison was made between the accuracy of the RMS parameter for the presented research methodology and for a single GPS SPP solution separately for the Thales Mobile Mapper and Topcon HiPerPro receivers. The accuracy of the RMS of a single SPP solution for each GPS receiver was determined according to Equation (8). The results of the RMS parameters along the X, Y, and Z-axes using the proposed methodology of measurement weights are shown in Table 1, while Figure 7 shows the results of the comparison of the RMS error values along the X-axis using both proposed the measurement weights solution (numbers 1 and 2) as well as the classical GPS SPP solution for Thales and Topcon (numbers 3 and 4, respectively). For the Thales Mobile Mapper, the RMS accuracy was 4.89 m and for the Topcon HiPerPro, it was 4.83 m. 

The classic solution for the SPP code method for the Thales Mobile Mapper and Topcon HiPerPro receivers was determined using the following mathematical formula [22,26]:

(9) l=d+c·(dtr−dts)+Ion+Trop+TGD+Rel+Mp

where l is the L1-C/A code measurement separately recorded by Thales Mobile Mapper and Topcon HiPerPro receivers; c is the speed of light; dtr is the receiver clock bias; dts is the satellite clock bias; Ion is the ionosphere correction; Trop is the troposphere correction; Rel is the relativistic effect; TGD is the Timing Group Delay; and Mp is the multipath effect.

The workflow of developing GPS observations for both satellite receivers in a stochastic process is presented in Section 3.

Comparing the RMS values, one can clearly see that the results obtained from the presented research method were better than the position solution from a single GPS receiver. Therefore, the accuracy of the position determination from the proposed method was better than from the position solution from the GPS SPP method. Table 2 shows the percentage changes of the increase in RMS accuracy for the developed methodology for the X component of the airplane 3D position. The percentage increase in the RMS accuracy improvement for the tested methodology is presented in the mathematical relation as follows:

(10) $$Ux\left[\%\right]=\{\begin{array}{c} \frac{(RMSdX_{w}-RMSdX_{Thales)}}{RMSdX_{Thales}}\cdot 100\%\\ \frac{(RMSdX_{w}-RMSdX_{Topcon}}{RMSdX_{Topcon}}\cdot 100\% \end{array}$$

where $\;RMSdX_{w}$ is the RMS error along X axis, based on Equation (8); $\;RMSdX_{Thales}$ is the RMS error along the X-axis for GPS SPP positioning for the Thales Mobile Mapper receiver; $\;RMSdX_{Topcon}$ is the RMS error along the X-axis for GPS SPP positioning for the Topcon HiPerPro receiver.

The calculation produced satisfactory results for parameter Ux. Consequently, the RMS positioning accuracy was increased by 2.5% and 3.2% when comparing the results of the GPS SPP positioning of Thales $\left(RMSdX_{Thales}\right)$ with $RMSdX_{w}$. In the case of Topcon, when comparing the results of the GPS SPP positioning ($RMSdX_{Topcon}$) with $RMSdX_{w}$ the positioning accuracy increased by 1.2% and 2.1%. Therefore, it can be said that the methodology presented in the paper is correct and the results are promising when considering the analysis of positioning accuracy of the aircraft along the X-axis. The improvement in accuracy along the X-axis was a small percentage, as it was about 1–3%. The effect of the accuracy improvement was therefore low along the X-axis. Although the accuracy along the X-axis was the lowest and was about 5 m, comparing the individual RMS parameters gave a small percentage effect. This was mainly due to the high RMS value. It can therefore be concluded that the larger the numerical values of the RMS, the percentage improvement in accuracy expressed by the Ux parameter gave smaller effects.

**Figure 8:**
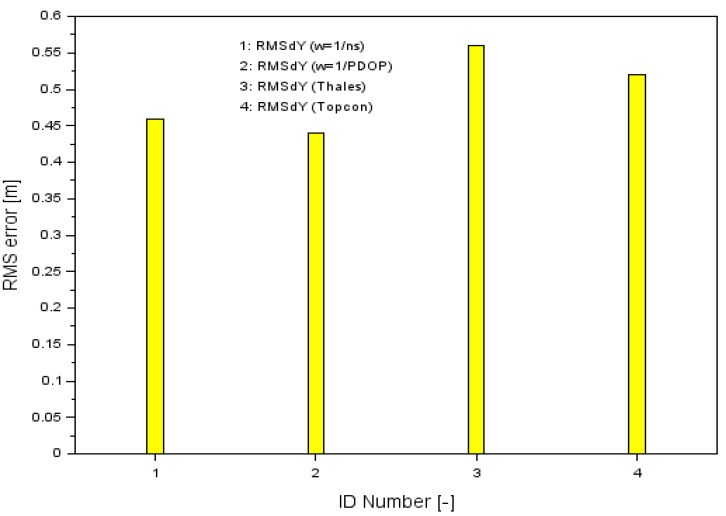
Comparison of RMS errors along the Y-axis.

The results of the RMS parameters along the Y-axis are shown in Table 1, while Figure 8 shows the results of the proposed measurement weights solution (numbers 1 and 2) along the Y-axis, and additionally the RMS error results for the SPP solution for single Thales and Topcon GPS receivers as sequence numbers 3 and 4, respectively. For the Thales Mobile Mapper receiver, the RMS accuracy was 0.56 m, while for the Topcon HiPerPro receiver it was 0.52 m. 

Comparing the RMS values in Figure 8, one can see that the errors obtained from the presented calculation method were smaller than the position solution from individual GPS receivers. Therefore, the accuracy of the position determination for the Y component of the proposed method was higher than that of the position solution from a single GPS receiver. Table 3 shows the percentage changes in the increase in RMS accuracy for the developed methodology. The percentage increase in RMS accuracy for the Y component for the proposed calculation methodology is derived using the mathematical relation as below:

(11) $$Uy\left[\%\right]=\{\begin{array}{c} \frac{\left(RMSdY_{w}-RMSdY_{Thales}\right)}{RMSdY_{Thales}}\cdot 100\%\\ \frac{\left(RMSdY_{w}-RMSdY_{Topcon}\right)}{RMSdY_{Topcon}}\cdot 100\% \end{array}$$

where$\;RMSdY_{w}$ is the RMS error along Y axis, based on Equation (8);$\;RMSdY_{Thales}$ is the RMS error along the Y-axis for GPS SPP positioning for the Thales Mobile Mapper receiver; and$\;RMSdY_{Topcon}$ is the RMS error along the Y-axis for GPS SPP positioning for the Topcon HiPerPro receiver.

The calculation produced optimistic results of the Uy parameter. Accordingly, the positioning accuracy of RMS was increased by 17.8% and 21.4% when comparing the results of $RMSdY_{w}$ with $RMSdY_{Thales}$, whereas when comparing the results of $RMSdY_{w}$ with $RMSdY_{Topcon}$, positioning accuracy increased by 11.5% and 15.4%. It can therefore be said that the calculation methodology presented in the paper is correct when considering the analysis of positioning accuracy of the aircraft along the Y-axis.

**Figure 9:**
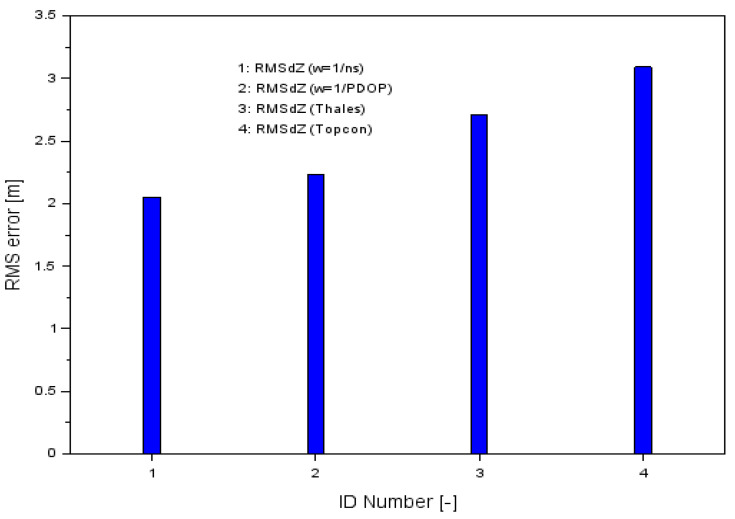
Comparison of the RMS errors along the Z-axis.

The outcomes of the RMS parameters along the Z-axis are presented in Table 1, while Figure 9 shows the results of the proposed measurement weights solution (numbers 1 and 2) along the Z-axis and the RMS error results for the SPP solution for single GPS receivers as sequence numbers 3 and 4 for the Thales and Topcon receivers, respectively. For the Thales Mobile Mapper, the RMS accuracy was 2.71 m and for the Topcon HiPerPro, it was 3.09 m. 

When comparing the RMS values in Figure 9, one can see that the errors obtained from the presented method were smaller than the position solution from a single GPS receiver. Therefore, the accuracy of the position determination for the Z component of the proposed method was better than that of the position solution from a single GPS receiver. Table 4 shows the percentage changes in the increase in RMS accuracy for the developed methodology along the Z-axis. The percentage change of RMS accuracy change for the presented methodology is given according to the mathematical relation as below:

(12) $$Uz\left[\%\right]=\{\begin{array}{c} \frac{(RMSdZ_{w}-RMSdZ_{Thales)}}{RMSdZ_{Thales}}\cdot 100\%\\ \frac{(RMSdZ_{w}-RMSdZ_{Topcon}}{RMSdZ_{Topcon}}\cdot 100\% \end{array}$$

where$\;RMSdZ_{w}$ is the RMS error along Z axis, based on Equation (8);$\;RMSdZ_{Thales}$ is the RMS error along Z-axis for GPS SPP positioning for Thales Mobile Mapper receiver;$\;RMSdZ_{Topcon}$ is the RMS error along the Z-axis for GPS SPP positioning for the Topcon HiPerPro receiver.

As a result of the calculations, very reliable and credible values of the Uz parameter were obtained. The positioning accuracy of RMS was increased by 17.7% and 24.5% when comparing the results of $RMSdZ_{w}$ with $RMSdZ_{Thales}$. When comparing the results of $RMSdZ_{w}$ with$\;RMSdZ_{Topcon}$, the positioning accuracy increased by 27.8% and 33.7%. It can be seen that for RMS errors along the Z-axis, there was a significant increase in positioning accuracy of the aircraft for the analyzed methodology.

The presented research methodology indicates the possibility of increasing the accuracy of GNSS aircraft positioning using multi-receivers. The problem of the low accuracy of GNSS positioning in aviation has been evident in many research works published in the past. The problem of low accuracy has been particularly noticeable in the case of using several GPS receivers in aviation tests in Poland [16,17,18,19,20,21,23,24]. The calculation strategy for determining the resultant position of an aircraft in air navigation presented in this paper could be effective and useful if applied in the above-mentioned aerial tests. It can be said that the measurement weighting strategy used in the paper is optimal and can be used in GNSS multi-receivers positioning in aviation. The obtained results clearly emphasize the effectiveness and efficiency of the presented numerical solution in air navigation.

In the final stage, to present an overall optimization effect, a comparison of the Cessna 172 resultant 3D position was made according to the formula:

(13) $$ds=\{\begin{array}{c} \sqrt{{\left(X_{GPS}^{m}-X_{ref}\right)}^{2}+{\left(Y_{GPS}^{m}-Y_{ref}\right)}^{2}+{\left(Z_{GPS}^{m}-Z_{ref}\right)}^{2}}\\ \sqrt{{\left(X_{Thales}-X_{ref}\right)}^{2}+{\left(Y_{Thales}-Y_{ref}\right)}^{2}+{\left(Z_{Thales}-Z_{ref}\right)}^{2}}\\ \sqrt{{\left(X_{Topcon}-X_{ref}\right)}^{2}+{\left(Y_{Topcon}-Y_{ref}\right)}^{2}+{\left(Z_{Topcon}-Z_{ref}\right)}^{2}} \end{array}$$

where ds is the displacement of the aircraft’s position relative to the reference trajectory; $\left(X_{Thales},Y_{Thales},Z_{Thales}\right)$ is the individual SPP solution for the Thales Mobile Mapper receiver; and $\left(X_{Topcon},Y_{Topcon},Z_{Topcon}\right)$ is the individual SPP solution for the Topcon HiPerPro receiver.

Table 5 shows the results of the 3D positional displacements of the aircraft as an arithmetic mean of the ds parameter. The ds parameter values for both weights were better than from an individual SPP solution for the Thales and Topcon receiver. The results of the ds parameter for the measurement weight $\left(w=\frac{1}{ns}\right)$ were improved by 2% compared to the SPP solution for the Thales receiver and by 13% compared to the SPP solution for the Topcon receiver. On the other hand, the results of the ds parameter for the measurement weight $\left(w=\frac{1}{PDOP}\right)$ were improved by 6% compared to the SPP solution for Thales and by 17% compared to the SPP solution for Topcon. The overall effect of accuracy improvement was clearly visible for the XYZ coordinates in 3D space. It can be concluded that weighing the measurements resulted in an improvement in the quality of flight trajectory in 3D space in the range of 2 to 17%.

## 6. Conclusions

This article presents a new calculation strategy for determining coordinates and increasing the accuracy of the GPS SPP code method of positioning in air navigation. In the paper, the weighted average method was used to improve the accuracy of the aircraft’s coordinates using the GPS SPP method. In this study, the measurement weights as a function of the number of GPS satellites being tracked and the PDOP value were applied. The weighted average model was proposed for solving the aircraft position expressed in XYZ geocentric coordinates. In the analyses, the real GPS navigation data from two on-board receivers, the Thales Mobile Mapper and Topcon HiPerPro, installed on the Cessna 172 aircraft were used. The research determined the accuracy of the proposed methodology by calculating the position errors and RMS error. The accuracy parameters were computed with reference to the precise reference position of the aircraft determined by the differential RTK-OTF technique. The effectiveness of the proposed solution was tested for a single SPP solution separately for both GPS receivers. When comparing the RMS error of the weighted average method to the regular GPS SPP method for the Thales Mobile Mapper receiver, it can be concluded that:

- The RMS improved by 2.5% and 3.2% using the weighted average model for the X component;
- The RMS improved by 17.8% and 21.4% by applying the weighted average model for the Y component; and
- The RMS improved by 17.7% and 24.5% by applying the weighted average model for the Z component.
- In the case of comparing the RMS error of the weighted average method to regular GPS SPP method for the Topcon HiPerPro receiver, it can be stated that:
- The RMS improved by 1.2% and 2.1% using the weighted average model for the X component;
- The RMS improved by 11.5% and 15.4% by applying the weighted average model for the Y component; and
- The RMS improved by 27.8% and 33.7% using the weighted average model for the Z component.

It should be pointed out that for the X and Y components, the best results were obtained when using a measurement weight as a function of the PDOP value. For the Z component, the best results were obtained when using a measurement weight as a function of the number of GPS satellites being tracked. 

To conclude, the test method proposed in the paper confirms the validity of using the weighted average model in precise aircraft positioning using the GPS SPP code method. It can be stated that the research methodology can be implemented and applied to air navigation. The proposed test method can be used along the entire flight path of the aircraft, from take-off to landing. Nevertheless, this method is of special importance during the approach and landing phase of the flight. Tests performed and completed in this way can be applied both at military and civil airports, for which GNSS approach procedures have been developed and implemented. Therefore, the applied calculation strategy would be especially useful for airports equipped with GNSS based approach cards. It should be noted that the presented results in the paper were from the weighted average model used for the speed range from 0 to 80 m/s [28]. The examination of the accuracy of the SPP code method in relation to the flight speed is an important aspect in determining the navigational parameters of the aircraft dynamics in air navigation. 

The authors plan to perform further aerial tests in the future in order to thoroughly examine the proposed method using other GNSS navigation systems and different quality GNSS satellite receivers. Such tests will allow us to fully check and verify the validity of the proposed calculation strategy for the GPS SPP code method in aviation.

## Figures and Tables

**Table 1:** The achieved results of the RMS error for tested measurement weights.

RMS Value	Weight $w=\frac{1}{ns}$	Weight $\;w=\frac{1}{PDOP}$
*RMSdX*	4.77 m	4.73 m
*RMSdY*	0.46 m	0.44 m
*RMSdZ*	2.05 m	2.23 m

**Table 2:** The relation between RMS error for the tested methodology along the X-axis.

*Ux* Value	Weight $w=\frac{1}{ns}$	Weight $\;w=\frac{1}{PDOP}$
*RMSdX_w_* vs. *RMSdX_Thales_*	2.5%	3.2%
*RMSdX_w_* vs. *RMSdX_Topcon_*	1.2%	2.1%

**Table 3:** The relation between RMS error for the tested methodology along the Y-axis.

*Uy* Value	Weight $w=\frac{1}{ns}$	Weight $\;w=\frac{1}{PDOP}$
*RMSdY_w_* vs. *RMSdY_Thales_*	17.8%	21.4%
*RMSdY_w_* vs. *RMSdY_Topcon_*	11.5%	15.4%

**Table 4:** The relation between the RMS error for the tested methodology along the Z-axis.

*Uz* Value	Weight $w=\frac{1}{ns}$	Weight $\;w=\frac{1}{PDOP}$
*RMSdZ_w_* vs. *RMSdZ_Thales_*	24.5%	17.7%
*RMSdZ_w_* vs. *RMSdZ_Topcon_*	33.7%	27.8%

**Table 5:** The results of ds parameter.

Arithmetic Mean of *ds* Value	Value [m]
*ds* (*weight* $w=\frac{1}{ns}$)	4.99
*ds* (*weight* $\;w=\frac{1}{PDOP}$)	4.80
*ds* (*Thales*)	5.09
*ds* (*Topcon*)	5.62
